# Glutaminase 1 Regulates Neuroinflammation After Cerebral Ischemia Through Enhancing Microglial Activation and Pro-Inflammatory Exosome Release

**DOI:** 10.3389/fimmu.2020.00161

**Published:** 2020-02-07

**Authors:** Ge Gao, Congcong Li, Jie Zhu, Yi Wang, Yunlong Huang, Shu Zhao, Shiyang Sheng, Yu Song, Chenhui Ji, Chunhong Li, Xiaoyu Yang, Ling Ye, Xinrui Qi, Yanyan Zhang, Xiaohuan Xia, Jialin C. Zheng

**Affiliations:** ^1^Center for Translational Neurodegeneration and Regenerative Therapy, Shanghai Tenth People's Hospital Affiliated to Tongji University School of Medicine, Shanghai, China; ^2^Departments of Pharmacology and Experimental Neuroscience, University of Nebraska Medical Center, Omaha, NE, United States; ^3^Collaborative Innovation Center for Brain Science, Tongji University, Shanghai, China

**Keywords:** focal cerebral ischemia, glutaminase 1, microglial activation, brain inflammation, glutaminase inhibitor, exosome

## Abstract

Cerebral ischemia induces a robust neuroinflammatory response that is largely mediated by the activation of CNS resident microglia. Activated microglia produce pro-inflammatory molecules to cause neuronal damage. Identifying regulators of microglial activation bears great potential in discovering promising candidates for neuroprotection post cerebral ischemia. Previous studies demonstrate abnormal elevation of glutaminase 1 (GLS1) in microglia in chronic CNS disorders including Alzheimer's disease and HIV-associated neurocognitive disorders. Ectopic expression of GLS1 induced microglia polarization into pro-inflammatory phenotype and exosome release *in vitro*. However, whether GLS1 is involved in neuroinflammation in acute brain injury remains unknown. Here, we observed activation of microglia, elevation of GLS1 expression, and accumulation of pro-inflammatory exosomes in rat brains 72 h post focal cerebral ischemia. Treatment with CB839, a glutaminase inhibitor, reversed ischemia-induced microglial activation, inflammatory response, and exosome release. Furthermore, we found that the application of exosome secretion inhibitor, GW4869, displayed similar anti-inflammatory effects to that of CB839, suggesting GLS1-mediated exosome release may play an important role in the formation of neuroinflammatory microenvironment. Therefore, GLS1 may serve as a key mediator and promising target of neuroinflammatory response in cerebral ischemia.

## Introduction

Ischemic stroke (IS) is one of the most common causes of mortality and disability worldwide, and an important threat to patient health and quality of life (1). Microglia respond rapidly to brain injury including ischemia to produce excessive pro-inflammatory and neuro-modulatory cytokines such as tumor necrosis factor (TNF) and interleukin family members (2, 3). The elevation of pro-inflammatory cytokine levels in brain tissue and cerebrospinal fluid (CSF) exacerbates neuroinflammation, which eventually causes neuronal damage (4, 5). The activated innate immune response acts as a hallmark for the progression of cerebral ischemia injury in which microglia, the resident immune cells of the central nervous system (CNS), play a key role (6). Microglia are sensors to detect abnormal alterations in response to internal and external insults in the brain. The activation of microglia is the core of neuroinflammation, which forms the first line of defense for brain injury or disease (7, 8). It is widely known that microglial activation can be induced by extrinsic factors such as amyloid peptides (Aβ) accumulation, virus infection, and lipopolysaccharide (LPS) stimulation, but the intracellular mechanisms that mediate the activation process remain controversial (9, 10).

Glutaminase 1 (GLS1) is a mitochondrial enzyme that catalyzes the hydrolysis of glutamine to produce glutamate in the CNS (11). We previously observed abnormal elevation of GLS1 protein levels in activated microglia in the early AD mouse brains and the HIV-1-associated dementia patient brains (9, 10). The ectopic expression of GLS1 in neural stem cells and their differentiated cell causes chronic neuroinflammation, synaptic dysfunctions, and learning deficits in a transgenic mouse model (12). Besides, GLS1 induces pro-inflammatory response and exosome release of primary microglia (10). These exosomes exhibit pro-inflammatory effects when co-cultured with resting microglia. Thus, our previous studies demonstrated the strong association of inflammatory response with the GLS1 expression levels *in vitro* and in chronic brain injury models. However, it remains unknown whether GLS1 is involved in the formation of pro-inflammatory environment in acute brain damage and how this GLS1-induced neuroinflammatory cascade progresses *in vivo*.

In this study, we found that the expression levels of GLS1 were up-regulated in rat focal cerebral ischemic brains, positively correlated with increased expression of pro-inflammatory markers. The inhibition of GLS1 activity by CB839 reduced the infarction volume and alleviated inflammatory response post cerebral ischemia, demonstrating the essential roles of GLS1 in mediating the neuroinflammation *in vivo*. We next demonstrated that GLS1 modulated neuroinflammation via regulating the release of pro-inflammatory exosomes, ascertained by the reduction of exosome release after CB839 treatment and the alleviation of inflammatory response post exosome release inhibitor, GW4869, treatment. Our results provide the first direct evidence for the involvement of GLS1 in acute neuroinflammation in the brain.

## Materials and Methods

### Rats

Sprague Dawley rats were housed and bred in the Comparative Medicine animal facilities of Tongji University School of Medicine. All procedures were conducted according to protocols approved by the Institutional Animal Care and Use Committee of Tongji University School of Medicine [reference number: SYXK (HU) 2014-0026].

### Focal Cerebral Ischemia and Drug Administration

Rats were anesthetized with 10% chloralhydrate. Rectal temperature contralateral to middle cerebral artery occlusion (MCAO) were monitored continuously and maintained at 37.0 ± 0.5°C with heating pads. Ischemia was induced by MCAO with monofilament nylon suture. The right external carotid artery (ECA) was ligated with 6-0 silk suture, the right common carotid artery (CCA) and internal carotid artery (ICA) was isolated and separated from the vagus nerve. 0.26 mm monofilament nylon suture with a kink 19 ± 1 mm from its rounded polylysine-coated tip was introduced from CCA into ICA, to occlude the origin of the right middle cerebral artery (MCA). The nylon suture was fixed and the wound was closed. After 90 min of occlusion, the suture was removed to allow reperfusion. Sham-operated rats underwent identical surgery except that the suture was not inserted. CB839 and GW4869 were administrated 2 h post reperfusion intraperitoneally. Seven days after reperfusion, rats were anesthetized and perfused with 4% paraformaldehyde. Brain tissues were removed and sectioned for immunohistochemical analysis.

### TTC Assessment of Infarct Size

Twenty rats were used for assessing infarct size (5 rats for each group). The rats were killed at 7 days after MCAO and drug treatment. The brains were rapidly removed, frozen at −20°C for 30 min. Two millimeters of thick coronal sections of the brain were then cut in a rat brain matrix starting at the frontal pole. The sections were incubated with 2% triphenyltetrazolium chloride (TTC) in dark for 30 min at 37°C. After this, sections were fixed in 4% buffered formaldehyde solution for 20 min. The cerebral infarct area was outlined in white in sham and experimental rats. Infarct areas in each section were measured using Image J software. Cerebral infarct volume was measured as the percentage of the total contralateral hemisphere.

### Protein Extraction and Western Blot

Rats were euthanized and their brains were removed and homogenized by a homogenizer in the M-PER Protein Extraction Buffer (Pierce) containing a protease inhibitor cocktail (Sigma). Protein concentrations were determined with a BCA Protein Assay Kit (Pierce). Proteins (5–10 μg) from tissue lysates or proteins (20–30 μg) from cell lysates were separated by sodium dodecyl sulfate-polyacrylamide gel electrophoresis (SDS-PAGE) and electrophoretic transferred to polyvinyldifluoridene membranes (Millipore and Bio-Rad). Proteins were treated with purified primary antibodies for CD86 (rabbit, cat#ab86392, Abcam, 1:1,000), GLS1 (rabbit, cat#ab156876, Abcam, 1:1,000), CD206 (mouse, cat#AF2535, R&D Systems, 1:500), CD9 (rabbit, cat#ab223052, Abcam, 1:1,000), Flotillin-1 (mouse, cat#610820, BD Biosciences, 1:1,000), TNFα (rabbit, cat#ab6671, Abcam, 1:1,000), Cox2 (rabbit, cat#ab15191, Abcam, 1:100), or β-actin (Sigma) overnight at 4°C followed by a horseradish peroxidase-linked secondary anti-rabbit or anti-mouse antibody (Cell Signaling Technologies, 1:10,000). Antigen-antibody complexes were visualized by Pierce ECL Western Blotting Substrate (Thermo Fisher Scientific, Waltham, MA). For data quantification, films were scanned with a CanonScan 9950F scanner; the acquired images were then analyzed on a Macintosh computer using the free public domain NIH ImageJ program (at http://rsb.info.nih.gov/nih-image/).

### Immunocytochemistry

Immunocytochemistry was done as previously described (13). Briefly, tissue sections were fixed in 4% paraformaldehyde (Sigma) for 15 min at room temperature (RT), washed 3 times with PBS (Fisher), and incubated with permeabilizing and blocking buffer containing 5% goat serum (Vector Laboratories) and 0.2% Triton X-100 (Bio-Rad) in PBS for 1 h at RT. Fixed tissue sections were incubated with primary antibody for GLS1 (rabbit, cat#ab156876, Abcam, 1:1,000) or Iba1 (goat, cat# ab5076, Abcam, 1:500) overnight at 4°C. The next day, tissue sections were washed with PBS and incubated with secondary antibodies (Molecular Probes) for 1 h at RT. Samples were mounted using Vecta-Shield (Vector Laboratories). Images were taken using a Zeiss AX10 fluorescence microscope accompanied with ZEN 2.3 (blue edition) software.

### Isolation of Exosomes

Exosomes were isolated from brain extracellular spaces as previously described (14). Fresh rat brains were dissected and treated with 20 units/ml papain (Worthington) in Hibernate E solution (6 ml/brain; BrainBits, Springfield, IL) for 15 min at 37°C. The brain tissue was gently homogenized in 2 volumes (12 ml/brain) of cold Hibernate E solution. The brain homogenate was sequentially filtered through a 40 μm mesh filter (BD Biosciences) and a 0.2 μm syringe filter (Thermo Scientific). The filtrate was centrifuged at 300× g for 10 min, then at 3,000× g for 20 min, and 10,000× g for 30 min. Exosomes were collected through ultracentrifugation at 100,000× g for 2 h. All centrifugation steps were performed at 4°C.

### Nanoparticle Tracking Analysis (NTA)

The size and number of exosomes isolated from brain extracellular spaces were determined as previously described (10). Briefly, 20 μl exosome suspension was diluted into 1 ml solution and then used for NanoSight analysis. NTA was done on NanoSight NS300 system (Malvern Instruments, UK) with a sCMOS camera. The conditions of the measurements were set at 25°C, 1 cP viscosity, 25 s per capture frame and 60 s for measurement time. Three individual measurements were performed for measuring sizes and concentrations of exosomes.

### Statistical Analyses

Data from two groups were evaluated statistically by two-tailed, unpaired student *t*-test. Data were shown as mean ± s.d., and significance was determined as *p* < 0.05.

## Results

### Focal Cerebral Ischemia Induces Microglial Activation and Neuroinflammation

**Figure 1:**
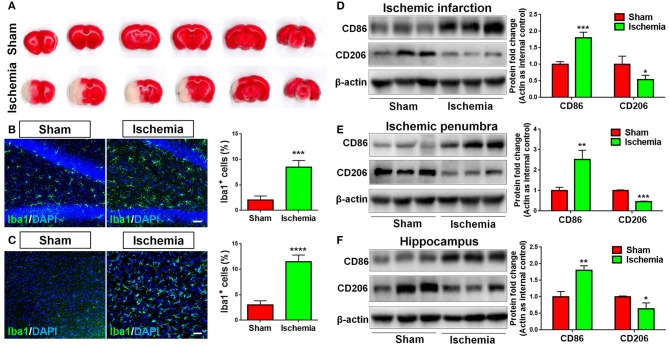
Focal cerebral ischemia induces neuroinflammation. **(A)** Serial coronal slices of sham-operated and MCAO rat brains. TTC staining showed red healthy zones and pale infarcted regions. **(B,C)** Focal cerebral ischemic brains and their sham controls were removed after intracranial perfusion and prepared for immunofluorescence staining. Representative pictures of Iba1 immunoreactivities in the hippocampus **(B)** and cortex **(C)** of focal cerebral ischemic rat brain sections and control rat brain sections were shown. Proportions of cells with Iba1 immunoreactivities were given on the right panel. **(D–F)** Representative blot and quantification of CD86 and CD206 protein expression levels in ischemic infarction **(D)**, ischemic penumbra **(E)**, and hippocampus **(F)**. Scale bar: 20 μm. Western blot data were normalized to β-actin and presented as fold change compared with those in sham rat brain. Error bars denote s.d. from triplicate measurements. *p < 0.05, **p < 0.01, ***p < 0.001, and ****p < 0.0001 by two-tailed *t*-test (*n* = 9).

To explore whether focal cerebral ischemia induces microglial activation, neuroinflammation, rats were subjected to MCAO. MCAO was validated by TTC staining, where cortical infarction could be observed 7 days post MCAO (Figure 1A). Immunohistochemical analysis revealed more Iba1^+^ cells in the hippocampus (Figure 1B) and cortex (Figure 1C) of focal cerebral ischemic brain sections, compared with sham control, revealing the activation of microglia in ischemic brain. The levels of the pro-inflammatory marker CD86 and that of anti-inflammatory marker CD206 were used as indicators of neuroinflammation. We found that MCAO significantly enhanced the expression of CD86 and inhibited that of CD206, in ischemic infarction (Figure 1D), ischemic penumbra (Figure 1E), and hippocampus (Figure 1F) in the ischemic brain hemisphere, suggesting neuroinflammation is induced by focal cerebral ischemia.

### GLS1 Expression Is Elevated in Focal Cerebral Ischemic

**Figure 2:**
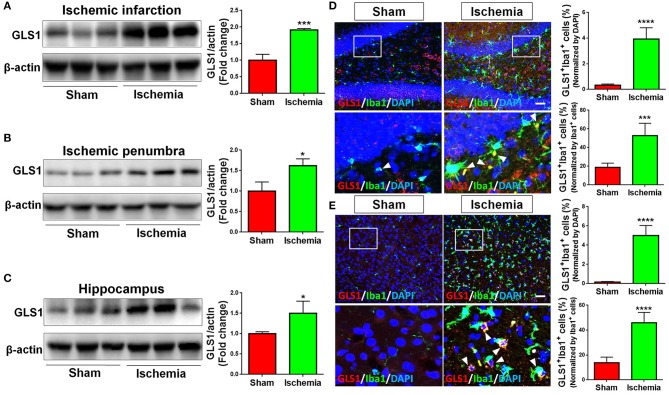
Up-regulation of GLS1 in focal cerebral ischemic brain. **(A–C)** Representative blot and quantification of GLS1 protein expression levels in ischemic infarction **(A)**, ischemic penumbra **(B)**, and hippocampus **(C)**. **(D,E)** Focal cerebral ischemic brains and their sham controls were removed after intracranial perfusion and prepared for immunofluorescence staining. Representative pictures of GLS1 and Iba1 immunoreactivities in the hippocampus **(D)** and cortex **(E)** of focal cerebral ischemic rat brain sections and control rat brain sections were shown. Images at the lower panels were high-magnification images of the corresponding small box area from the upper panels. Quantification of Iba1^+^/GLS1^+^ cells among total cells or Iba1^+^ cells co-expressing GLS1 immunoreactivities was provided on the right panel. Scale bar: 100 μm. Data were normalized to β-actin and presented as fold change compared with those in sham rat brain. Error bars denote s.d. from triplicate measurements. *p < 0.05, ***p < 0.001, and ****p < 0.0001 by two-tailed *t*-test (*n* = 9).

The expression of GLS1 was determined by western blot. The abnormal elevation of GLS1 was observed in all tested brain regions post cerebral ischemia, displaying a positive correlation with the increase of pro-inflammatory microglial phenotype (Figures 2A–C). More importantly, GLS1 was found evidently co-localized with Iba1^+^ microglia in the hippocampus (Figure 2D) and cortex (Figure 2E) of focal cerebral ischemic brain sections. Quantification results further suggested an increase of the proportions of GLS1^+^/Iba1^+^ cells in total brain cells and activated microglia. Thus, these data demonstrate an elevation of GLS1 in microglia after MCAO, implying for the involvement of GLS1 in neuroinflammation.

### GLS1 Mediates Neuroinflammation in Focal Cerebral Ischemic Brain

**Figure 3:**
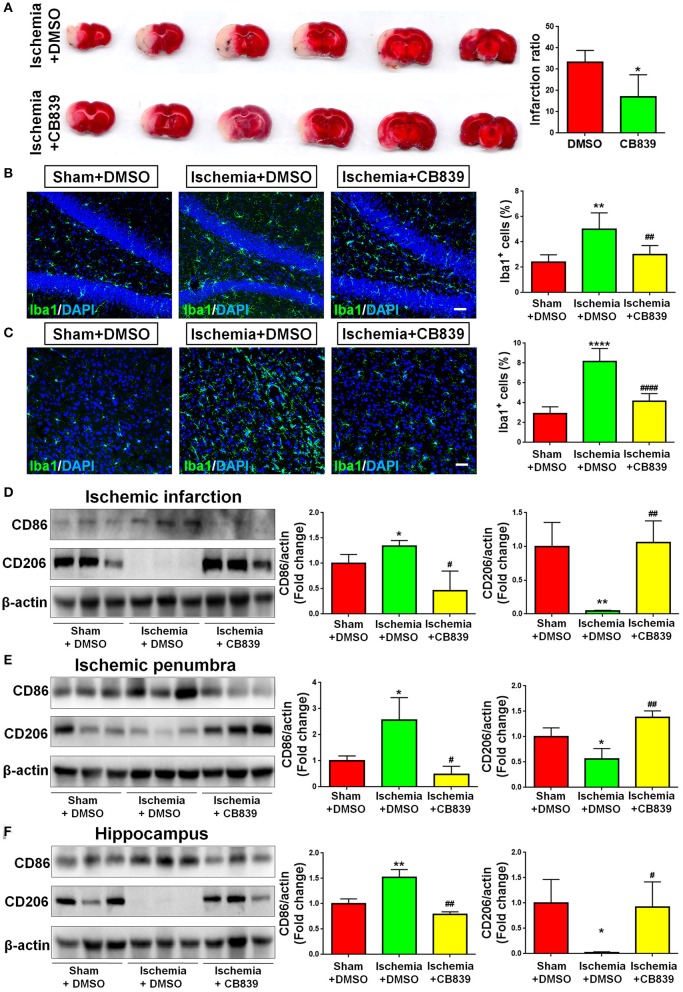
GLS1 inhibition by CB839 alleviates neuroinflammation. **(A)** TTC staining of rat brain serial coronal slices in sham, ischemia, and ischemia with CB839 treatment groups. Infarction volume is given on the right panel. **(B,C)** Focal cerebral ischemic brains treat with or without CB839 and their sham controls were removed after intracranial perfusion and prepared for immunofluorescence staining. Representative pictures of Iba1 expression in the hippocampus **(B)** and cortex **(C)** of all three groups were shown. Proportions of cells with Iba1 immunoreactivities were given on the right panel. **(D–F)** Representative blot and quantification of CD86 and CD206 protein expression levels in ischemic infarction **(D)**, ischemic penumbra **(E)**, and hippocampus **(F)**. Scale bar: 20 μm. Western blot data were normalized to β-actin and presented as fold change compared with those in sham rat brain. Error bars denote s.d. from triplicate measurements. *p < 0.05, **p < 0.01, and ****p < 0.0001 by two-tailed *t*-test (*n* = 9). ^#^*p* < 0.05, ^*##*^*p* < 0.01, and ^*####*^*p* < 0.0001 vs. the ischemia with DMSO treatment group by two-tailed *t*-test (*n* = 9).

In order to determine whether GLS1 is involved in microglial activation and neuroinflammation after MCAO, we administrated CB839, a commonly used GLS1 inhibitor, into MCAO rats intraperitoneally 2 h after surgery. After seven days of continuous CD839 administration, rats were sacrificed and postmortem TTC staining was performed (Figure 3A). The infarction ratio is significantly decreased in CB839 treatment groups vs. controls, suggesting the neuroprotective effects of CB839 by reducing infarction volume. Immunohistochemical analysis revealed that CB839 treatment reversed the positive effects of MCAO on microglial activation, ascertained by the reduction of cells expressing Iba1 immunoreactivities in both cortex (Figure 3B) and hippocampus (Figure 3C) of MCAO rat brain after 7 days CB839 intraperitoneal administration, compared with MCAO with DMSO treatment group. Protein analyses revealed that the expression of CD86 decreased and that of CD206 increased in the infarction region of CB839-injected MCAO rat brain, compared with MCAO group (Figure 3D). Similar expression patterns were observed in ischemic penumbra (Figure 3E) and hippocampus (Figure 3F), when comparing CB839-injected MCAO group with corresponding control group. Thus, our findings demonstrate that GLS1 is involved in the activation of microglia, formation of neuroinflammation, and the consequent neuronal damage in focal cerebral ischemic brains.

### Focal Cerebral Ischemia Induces Pro-Inflammatory Exosome Release

**Figure 4:**
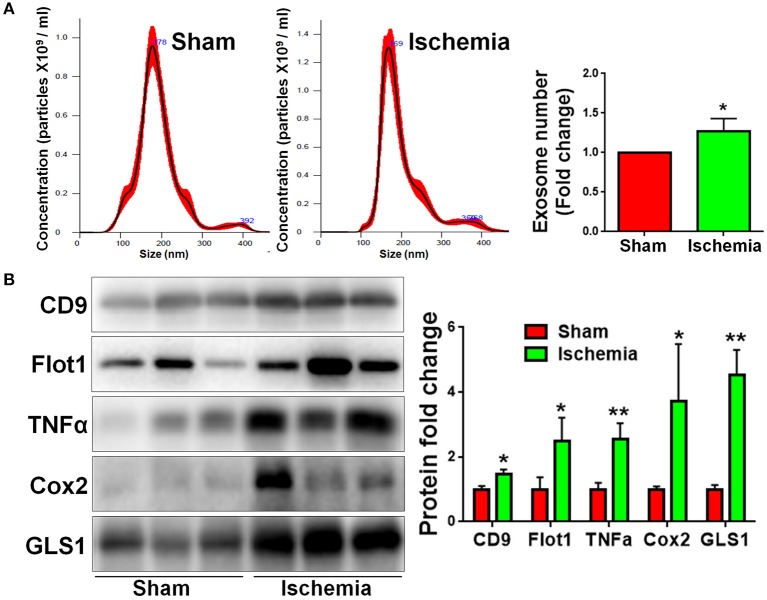
Focal cerebral ischemia induces exosome release. Exosomes were collected from ischemic and sham brain tissues for NTA **(A)** and western blot **(B)** analyses. **(A)** The concentration of exosome suspension was determined by NTA. **(B)** Representative immunoblots of CD9, Flot1, TNFα, Cox2, and GLS1 were shown along with quantifications of protein expression levels. Western blot data were presented as fold change compared with those in sham rat brains. Error bars denote s.d. from triplicate measurements. *p < 0.05 and **p < 0.01 by two-tailed *t*-test (*n* = 9).

We previously found that exosomes play an essential role in the activation of microglia and the formation of pro-inflammatory microenvironment *in vitro* (10, 15). To understand the involvement of exosomes in post-ischemia neuroinflammation *in vivo*, we firstly examined whether or not the release of exosomes are altered post focal cerebral ischemia. Exosomes were collected from right hemispherical extracellular spaces of individual sham and ischemic rats and suspended in constant volume. NTA revealed that, though the exosome sizes were similar between sham and ischemia groups, the concentrations of exosome were significantly higher in ischemia group compared to the sham control (Figure 4A). Consistent with the data on NTA, western blot analysis demonstrated that the expression levels of exosome markers CD9 and Flot1 were significant higher in ischemia group, compared to sham control, confirming an increase of exosome release post ischemia (Figure 4B). We also tested the contents of exosomes released from sham and ischemia groups. Quantitative results of western blot demonstrated that the levels of TNFα, a pro-inflammatory cytokines, and cyclooxygenase-2 (Cox2), a key enzyme that mediates inflammatory signaling, were significantly higher in ischemia group vs. control. Interestingly, we found that GLS1 levels in ischemic brain derived exosomes were higher than that in sham brain derived exosomes. Thus, our observations suggested that focal cerebral ischemia enhanced the release of exosomes and the loading of pro-inflammatory molecules in exosomes.

### GLS1 Mediates Pro-Inflammatory Exosome Release

**Figure 5:**
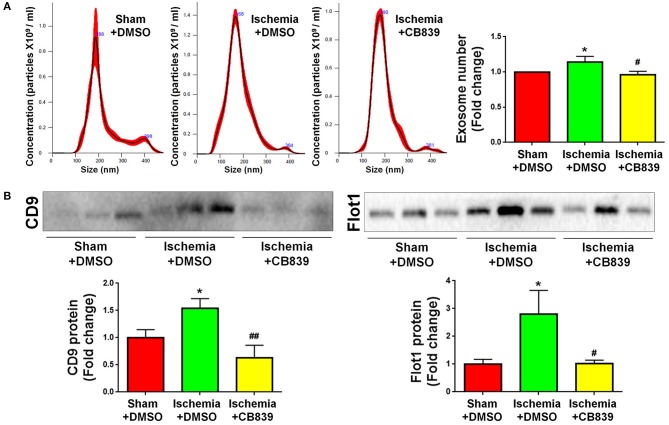
GLS1 inhibition by CB839 suppresses exosome release. Seven days post either DMSO or CB839 administration, exosomes were collected from ischemic and sham brain extracellular spaces for NTA **(A)** and western blot **(B)** analyses. **(A)** The concentration of exosome suspension was determined by NTA. **(B)** Representative immunoblots of CD9 and Flot1 were shown along with quantifications of protein expression levels. Data were presented as fold change compared with those in sham rat brains. Error bars denote s.d. from triplicate measurements. *p < 0.05 by two-tailed *t*-test (*n* = 9). ^#^*p* < 0.05 and ^*##*^*p* < 0.01 vs. the ischemia with DMSO treatment group by two-tailed *t*-test (*n* = 9).

Because our previous findings indicate that GLS1 is a key enzyme in exosomes release of glial cells including microglia and astrocytes *in vitro* (16, 17), we examined whether or not the alteration of exosome release in focal cerebral ischemic brains is mediated by GLS1. NTA demonstrated that GLS1 inhibition by CB839 administration significantly reduced the concentration of exosome in ischemic rat brains vs. the DMSO-treated ischemia group (Figure 5A). Western blot analysis also revealed that CB839 administration significantly suppressed the release of exosomes, ascertained by the reduction of CD9 and Flot1 protein levels in ischemia with CB839 treatment group, compared with that in ischemia with DMSO treatment group (Figure 5B). Hence, our results suggested the important role of GLS1 on exosome release *in vivo*.

### The Inhibition of Exosome Release by GW4869 Alleviates Neuroinflammation in Focal Cerebral Ischemic Brain

**Figure 6:**
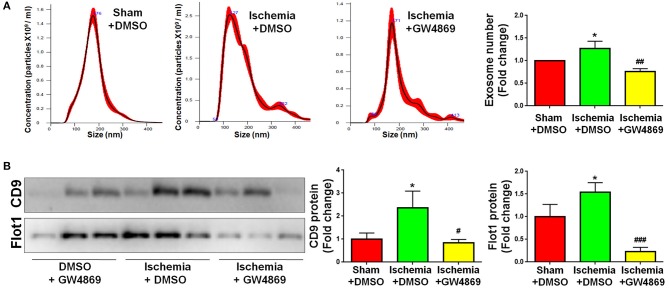
GW4869 administration inhibits exosome release. Seven days post either DMSO or GW4869 administration, exosomes were collected from ischemic and sham brain extracellular spaces for NTA **(A)** and western blot **(B)** analyses. **(A)** The concentration of exosome suspension was determined by NTA. **(B)** Representative immunoblots of CD9 and Flot1 were shown along with quantifications of protein expression levels. Western blot data were presented as fold change compared with those in sham rat brains. Error bars denote s.d. from triplicate measurements. *p < 0.05 by two-tailed *t*-test (*n* = 9). ^#^*p* < 0.05, ^*##*^*p* < 0.01, and ^*###*^*p* < 0.001 vs. the ischemia with DMSO treatment group by two-tailed *t*-test (*n* = 9).

**Figure 7:**
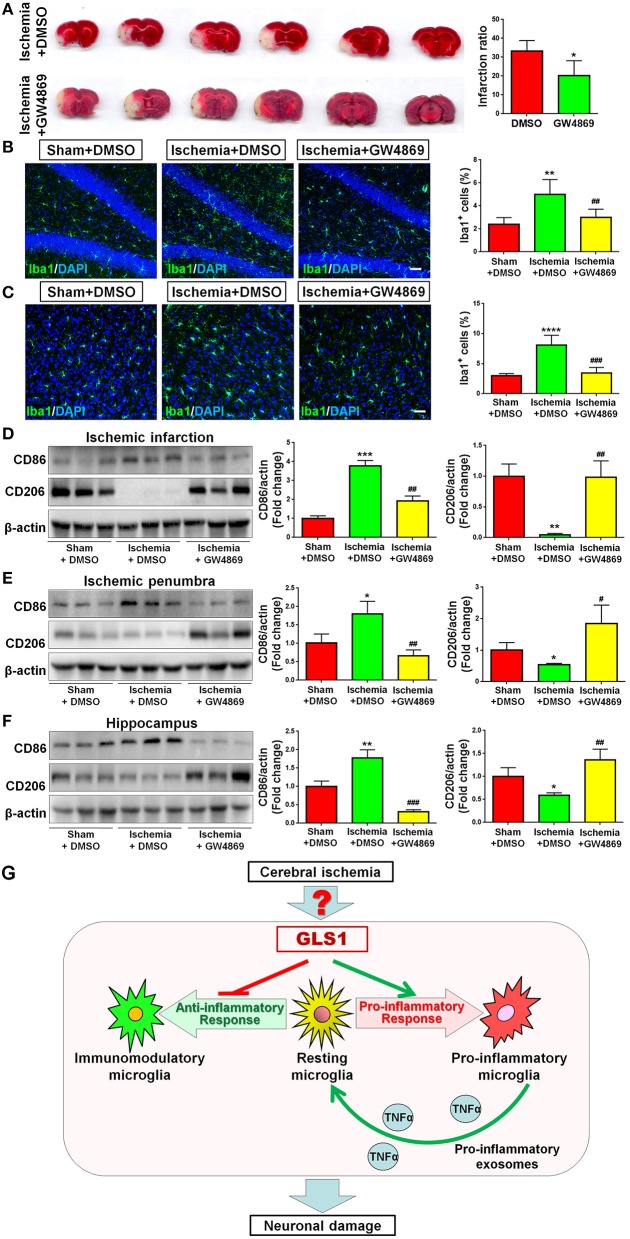
Exosome release inhibition by GW4869 alleviates neuroinflammation. **(A)** TTC staining of rat brain serial coronal slices in sham, ischemia, and ischemia with GW4869 treatment groups. Infarction volume is given on the right panel. **(B,C)** Focal cerebral ischemic brains treated with or without GW48639 and their sham controls were removed after intracranial perfusion and prepared for immunofluorescence staining. Representative pictures of Iba1 expression in the hippocampus **(B)** and cortex **(C)** of all three groups were shown. Proportions of cells with Iba1 immunoreactivities were given on the right panel. **(D–F)** Representative blot and quantification of CD86 and CD206 protein expression levels in ischemic infarction **(D)**, ischemic penumbra **(E)**, and hippocampus **(F)**. **(G)** A schematic representation of GLS1-mediated neuroinflammation: the up-regulation of GLS1 expression in focal cerebral ischemic brain shifts the resting microglia toward pro-inflammatory phenotype, but against immunomodulatory one. GLS1 then enhances the release of pro-inflammatory exosomes from pro-inflammatory microglia, leading to the formation of inflammatory microenvironment, which induces neuronal damage. Scale bar: 20 μm. Western blot data were normalized to β-actin and presented as fold change compared with those in sham rat brain. Error bars denote s.d. from triplicate measurements. *p < 0.05, **p < 0.01, ***p < 0.001, and ****p < 0.0001 by two-tailed *t*-test (*n* = 9). ^#^*p* < 0.05, ^*##*^*p* < 0.01, and ^*###*^*p* < 0.001 vs. the ischemia with DMSO treatment group by two-tailed *t*-test (*n* = 9).

As GLS1 associates with the inflammatory response and pro-inflammatory exosome release in focal cerebral ischemic brains, we hypothesized that GLS1 might regulate post-ischemia neuroinflammation via exosome release. To test our premise, we administrated GW4869, a neutral sphingomyelinase inhibitor known to suppress exosome release, into MCAO rats intraperitoneally 2 h after surgery. Rats were sacrificed after 7 days of continuous GW4869 administration. We firstly tested the effects of GW4869 treatment on exosome release *in vivo*. NTA suggested that GW4869 administration significantly reduced the concentration of exosome in ischemic rat brains vs. the ischemia with DMSO treatment control (Figure 6A). Similarly, western blot analysis revealed lower expression levels of CD9 and Flot1 in exosomes collected from GW4869-treated ischemic hemispheres, compared with DMSO-treated ischemic ones, suggesting less exosomes were released in rat brain extracellular spaces post GW4869 administration (Figure 6B). The cerebral infarction was assessed by TTC staining, which displayed a significant reduction in the infarction volume in GW4869-injected group vs. its corresponding controls (Figure 7A). More importantly, we observed a reduction of cells expressing Iba1 immunoreactivities in both cortex and hippocampus of MCAO rat brain after seven days GW4869 intraperitoneal administration, compared with MCAO with DMSO treatment group, suggesting that GW4869 administration achieved a similar effect as CB839-induced GLS1 inhibition in reversing microglial activation (Figures 7B,C). Furthermore, GW4869 administration also alleviates neuroinflammation post cerebral ischemia, ascertained by the decrease of CD86 and increase of CD206 in all test brain regions in GW4869-treated groups vs. DMSO-treated groups (Figures 7D–F). Thus, our studies demonstrated the essential roles of exosomes in the induction of microglial activation and inflammatory response post MCAO, implying GLS1-mediated pro-inflammatory exosome release as a novel mechanism in post-ischemia neuroinflammation and brain injury.

## Discussion

Neuroinflammation is one of the key pathophysiological features of IS, which involves activation of glial cells in the CNS as well as production and release of large numbers of inflammatory molecules (18, 19). Blockade of inflammation is considered a possible approach to the therapy of cerebral ischemia (20). For example, inhibition of microglial activation by various molecules, such as minocycline (21) and reparixin (20), was shown to result in less neuronal damage. Microglia are the first line of defenders and inflammatory responders against pathogens, wounds, and injuries. They contribute to ischemic damage as being capable of expressing potentially harmful molecules including pro-inflammatory cytokines (7, 8, 10, 19). Thus, to identify the cell extrinsic and intrinsic factors that regulate microglial activation are key to prevent or alleviate neuroinflammation timely and efficiently.

In the current study, we investigated the effects of GLS1 on inflammatory response to brain injury *in vivo*, using focal cerebral ischemia as an acute brain injury model. We demonstrated a significantly heightened expression of GLS1 in focal cerebral ischemic brain tissues, matching with the observations of Newcomb et al. (22). The GLS1 expression patterns are positively correlated with the elevation of pro-inflammatory molecules. The inhibition of GLS1 activity by CB839 administration reduced infarction volume and alleviated inflammation. Importantly, the release of pro-inflammatory exosomes was enhanced post ischemia, which, can be reversed by GLS1 inhibitor treatment. Furthermore, the inhibition of exosome release by GW4869 achieved similar anti-inflammatory effects as CB839. In all, these results demonstrate a causal effect of GLS1 overexpression on microglia activation in ischemic brain, via increased release of exosomes with pro-inflammatory content (Figure 7G).

GLS1, the primary enzyme responsible for the generation of glutamate in the CNS, is abundantly expressed in neurons (e.g., cortical neurons) (23–25). We, together with other groups, also observed GLS1 expression in glial cells including astroglia (26) and microglia (9), although with lower levels than that in neurons (27). Our previous studies suggested that HIV-1 infection induces GLS1 expression in microglia and macrophages, resulting in abnormal glutamate production (9, 28). Excessive glutamate causes excitatory neurotoxicity, ultimately leading to neuronal loss (9). Our recent studies reveal a critical role of GLS1 in microglial activation in chronic inflammation conditions, such as neurodegenerative diseases including Alzheimer's Disease (AD) (10) and HIV-1-associated neurocognitive disorders (HAND) (9). The heightened GLS1 expression was observed in activated microglia in the mouse brain tissues with early onset of AD and the brain tissues of HAND patients. The ectopic expression of GLS1 in primary cultured microglia led to the activation of microglia and the production of pro-inflammatory molecules (10). It matches with the reports that the inhibition of glutamine synthetase, an enzyme with opposite function of GLS1 in glutaminolysis, shifts of resting microglia to pro-inflammatory states (29, 30). Interestingly, we found that overexpression of GLS1 in microglia did not facilitate glutamate secretion and excessive extracellular glutamate did not induce microglial activation, suggesting that GLS1 does not regulate microglial activation through releasing glutamate (10). It is somehow expected since microglia did not express any inotropic glutamate receptors, or metabotropic glutamate receptors except mGluR2/3, leaving them insensitive to extracellular glutamate (10). Hence, GLS1 presumably regulates microglial activation via intracellular mechanisms (23). A possible one is that GLS1, as a mitochondrial enzyme having a key role in cellular bioenergetics and metabolism, may control the generation of reactive oxygen species (ROS) via regulating the ratio of α-ketoglutarate and succinate, two metabolites downstream of glutaminolysis (31, 32). The production of ROS further induces microglial activation by exacerbating oxidative stress (33) and activating signaling pathways including HIFα and Nf-kb pathways (34).

Our current study suggests an essential pathogenic role of GLS1 in the formation of pro-inflammatory microenvironment post ischemia, presumably through regulating exosome release. Exosomes, a key intercellular communication mediator, have been detected at an elevated level in the CSF of patients with chronic neuroinflammation, such as AD, Parkinson's disease, and amyotrophic lateral sclerosis (35). Mechanisms regulating exosome release remain poorly investigated. Our previous studies reveal an increase in exosomes released from activated macrophages (17) and microglia (10) *in vitro*, which, can be regulated by GLS1. Reducing either the increased release of exosomes or the heightened expression of GLS1 significantly reversed the activation of primary cultured macrophages/microglia. The current study revealed that GLS1 overexpression promoted pro-inflammatory exosome release post ischemia. The inhibition of either GLS1 activity or exosome release display similar anti-inflammatory effects, providing *in vivo* evidence for the involvement of GLS1-mediated exosome release in neuroinflammation post ischemia. It is worth-noting that the treatment of CB839 and GW4869 may also inhibit exosome release from other cell types beside microglia. But since the majority of pro-inflammatory molecules are secreted by activated microglia, it is highly likely that pro-inflammatory cytokines packaged into exosomes are mainly produced and released by microglia. Thus, the blockage of exosome release prevented the spread of cytokines, generated by microglia and other cell types, in the brain and attenuated the initiation and progression of neuroinflammation *in vivo*. Besides, we also found that GLS1 could be transferred intercellularly via exosomes, ascertained by western blot analysis of brain-derived exosomes. These results match with our previous observations *in vitro* that HIV-1-infected macrophages and immune-activated microglia secret GLS1-containing exosomes (15). It is remain unknown that whether or not GLS1 in exosomes remains active. If so, the elevation of GLS1 levels in recipient cells may induce over-production of glutamate. Excessive intracellular glutamate causes abnormal cell metabolic changes such as ATP production (36) and ROS accumulation (31) in mitochondria, which leads to neuronal damage (37), microglial activation (38), and exosome release (17). And superfluous extracellular glutamate causes continuous hyperactivation of NMDA receptors in neurons that results in neurotoxicity (9). These results suggest that, except enhancing the selective loading of pro-inflammatory cytokines and miRNAs (10), GLS1 itself may serve as a harmful molecule in inducing neuroinflammation and other pathological events.

Due to the importance of GLS1 in neuroinflammation and neurotoxicity, the potential of clinical translation of GLS1 inhibitors (e.g., CB839, Bptes, JHU-083, etc.) in treating CNS disorders are currently under investigation. For instance, JHU-083, a prodrug of a classic GLS1 inhibitor L-DON, inhibits GLS1 activity in microglia to block inflammatory response and prevent depression-associated behaviors induced by chronic social defeat stress (39). Our study provided strong evidence that the inhibition of GLS1 by CB839 also alleviated neuroinflammation in acute brain injury, indicating bright prospects of GLS1 inhibitors in clinical application.

In all, the current study displayed a heightened expression of GLS1 in cerebral ischemic rat brain tissues, demonstrated a causal effect of GLS1 on inflammatory response and exosome release, and revealed the involvement of GLS1 in neuroinflammation and brain injury via altering the release and content of exosomes. These results identified a novel mechanism for the instigation of neuroinflammation and brain injury post IS, implying a potential candidate for the intervention and treatment of stroke.

## Data Availability Statement

All datasets generated for this study are included in the article/supplementary material.

## Ethics Statement

The animal study was reviewed and approved by The Institutional Animal Care and Use Committee of Tongji University School of Medicine [reference number: SYXK (HU) 2014-0026].

## Author Contributions

XX and JCZ conceived and designed the experiments. GG, CoL, JZ, SZ, SS, YS, CJ, ChL, and XY performed the experiments. GG, CoL, and XX analyzed the data. GG, CoL, JZ, XX, LY, XQ, YZ, and JCZ contributed reagents, materials, and analysis tools. XX, YW, YH, and JCZ wrote the paper.

### Conflict of Interest

The authors declare that the research was conducted in the absence of any commercial or financial relationships that could be construed as a potential conflict of interest.

## Abbreviations

Aβ: amyloid peptides
AD: Alzheimer's disease
CCA: common carotid artery
CNS: central nervous system
CSF: cerebrospinal fluid
ECA: external carotid artery
Flot1: flotillin-1
GLS1: glutaminase 1
HAND: HIV-1-associated neurocognitive disorders
ICA: internal carotid artery
IS: ischemic stroke
LPS: lipopolysaccharide
MCAO: middle cerebral artery occlusion
NTA: nanoparticle tracking analysis
ROS: reactive oxygen species
RT: room temperature
TNF: tumor necrosis factor
TTC: triphenyltetrazolium chloride.
